# App Use and Usability of a Barcode-Based Digital Platform to Augment COVID-19 Contact Tracing: Postpilot Survey and Paradata Analysis

**DOI:** 10.2196/25859

**Published:** 2021-03-26

**Authors:** Thomas Foster Scherr, Jenna Maria DeSousa, Carson Paige Moore, Austin Hardcastle, David Wilson Wright

**Affiliations:** 1 Department of Chemistry Vanderbilt University Nashville, TN United States

**Keywords:** contact tracing, COVID-19, mobile health, usability, app, usage, tracking, monitoring, survey, pilot

## Abstract

**Background:**

The COVID-19 pandemic has drastically changed life in the United States, as the country has recorded over 23 million cases and 383,000 deaths to date. In the leadup to widespread vaccine deployment, testing and surveillance are critical for detecting and stopping possible routes of transmission. Contact tracing has become an important surveillance measure to control COVID-19 in the United States, and mobile health interventions have found increased prominence in this space.

**Objective:**

The aim of this study was to investigate the use and usability of MyCOVIDKey, a mobile-based web app to assist COVID-19 contact tracing efforts, during the 6-week pilot period.

**Methods:**

A 6-week study was conducted on the Vanderbilt University campus in Nashville, Tennessee. The study participants, consisting primarily of graduate students, postdoctoral researchers, and faculty in the Chemistry Department at Vanderbilt University, were asked to use the MyCOVIDKey web app during the course of the study period. Paradata were collected as users engaged with the MyCOVIDKey web app. At the end of the study, all participants were asked to report on their user experience in a survey, and the results were analyzed in the context of the user paradata.

**Results:**

During the pilot period, 45 users enrolled in MyCOVIDKey. An analysis of their enrollment suggests that initial recruiting efforts were effective; however, participant recruitment and engagement efforts at the midpoint of the study were less effective. App use paralleled the number of users, indicating that incentives were useful for recruiting new users to sign up but did not result in users attempting to artificially inflate their use as a result of prize offers. Times to completion of key tasks were low, indicating that the main features of the app could be used quickly. Of the 45 users, 30 provided feedback through a postpilot survey, with 26 (58%) completing it in its entirety. The MyCOVIDKey app as a whole was rated 70.0 on the System Usability Scale, indicating that it performed above the accepted threshold for usability. When the key-in and self-assessment features were examined on their own, it was found that they individually crossed the same thresholds for acceptable usability but that the key-in feature had a higher margin for improvement.

**Conclusions:**

The MyCOVIDKey app was found overall to be a useful tool for COVID-19 contact tracing in a university setting. Most users suggested simple-to-implement improvements, such as replacing the web app framework with a native app format or changing the placement of the scanner within the app workflow. After these updates, this tool could be readily deployed and easily adapted to other settings across the country. The need for digital contact tracing tools is becoming increasingly apparent, particularly as COVID-19 case numbers continue to increase while more businesses begin to reopen.

## Introduction

The COVID-19 pandemic quickly evolved from localized transmission to broad and sustained community transmission across the United States [1]. Most states initially enacted stay-at-home orders to curb the spread of the virus, with schools and businesses shifting to virtual operations early in the pandemic [2-5]. As local restrictions have lifted, many schools and workplaces have implemented new changes that enable a safe return to work. These adjustments include masking and social distancing requirements as well the implementation of daily health checks [6-8].

Relative to many other infectious diseases, COVID-19 has a high degree of asymptomatic transmission [9,10]. An infected person may not know that they are infected, and if they are not engaging in good public health measures (eg, regular handwashing, mask wearing, social distancing), they may come into direct contact with other individuals and spread the virus. As such, there is a clear ceiling on the usefulness of symptom monitoring alone. It is widely recognized that broad testing and effective contact tracing are necessary to counter instances of unknowing transmission, particularly because widespread vaccine distribution has been slow to take hold.

Across the United States, contact tracing efforts have been implemented to varying extents [11-17]. When an individual is confirmed or suspected to be positive for COVID-19, contact tracers will interview that person and identify any close contacts that they have had during their infectious window. After building a list of potential contacts for each index case, tracers reach out to each of the contacts and let them know of their potential exposure, either helping them to locate nearby testing options or providing counseling on effective self-isolation.

In states and counties where there has been a rapid rise in cases, the need for contact tracing has often outpaced the ability to implement a rigorous surveillance system. This has presented an opportunity for developers of digital health tools, which were already increasing in use prior to the pandemic, to redirect their efforts to build contact tracing platforms [18-22]. Several digital contact tracing platforms have been described in the academic literature [23-25], and more are available through for-profit technology companies [20]. Although these tools use a variety of technologies, two of the most popular strategies are (1) continuous location monitoring and (2) observing Bluetooth interactions between devices. Due to the size and commercial motives of the developers of these platforms, they have been subject to intense scrutiny over potential privacy concerns regarding data ownership and use—even before they have been released. These apps, while simple and useful for contact tracing efforts, are viewed with skepticism by many people, who may not wish to share such granular personal data.

Due to the rapid emergence of the pandemic and the digital contact tracing tools that soon emerged in response, few formal studies have been performed to understand user priorities and improve usability. Modeling has been applied to examine contact tracing app acceptance rates [26], and frameworks have been proposed to evaluate the potential scalability of these apps [27]. Recently, a survey study identified the importance of enhancing perceived benefits and self-efficacy and also identified the perceived barrier of privacy concerns [28]. Although these findings are useful in the initial design of contact tracing platforms, none of these studies investigated specific existing apps.

**Figure 1:**
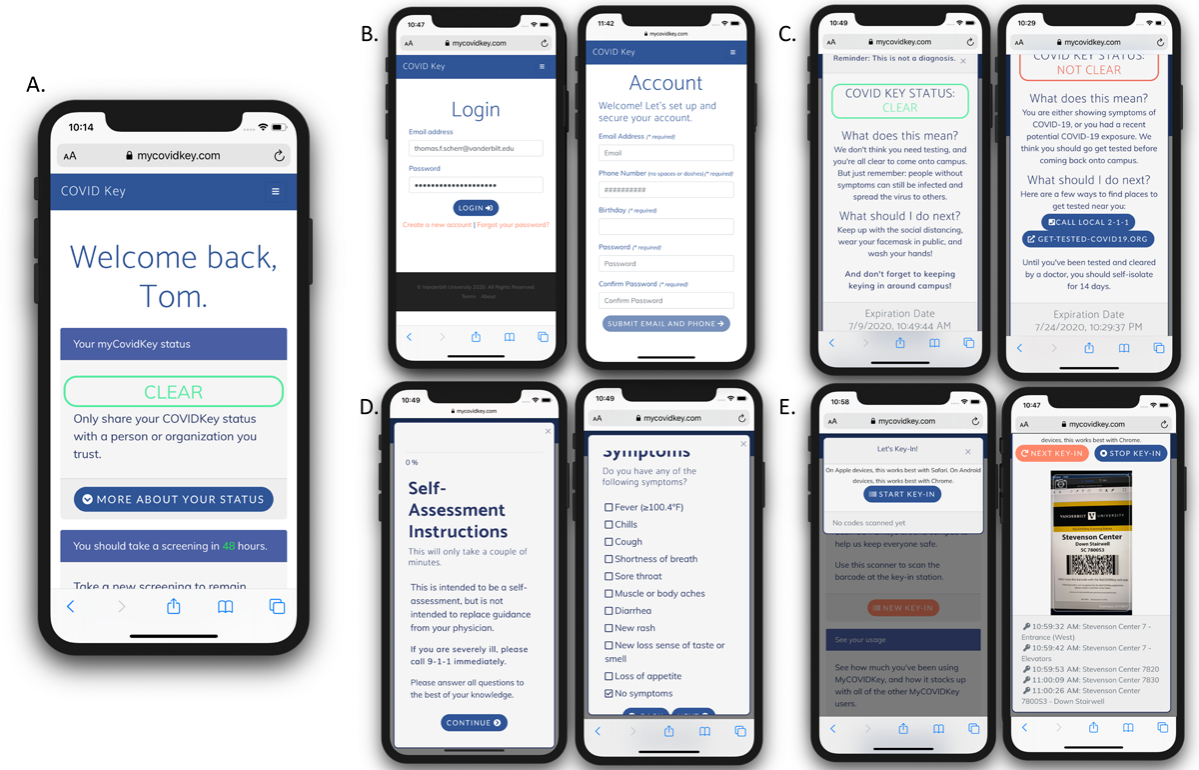
The main screens of the MyCOVIDKey web app: (A) the landing page, which presents a user’s status after a valid login and allows them to access self-assessments and key-ins; B) the login and account creation pages; (C) the screens for CLEAR (left) and NOT CLEAR (right) statuses; (D) the brief COVID-19 risk assessment; and (E) the key-in feature, which enables users to scan location-specific bar codes.

We previously described an alternative digital contact tracing tool, MyCOVIDKey, that is designed to supplement existing contact tracing infrastructure [29]. Our primary motivation was to develop a tool that would be less invasive while retaining efficacy. The software is a mobile-friendly web app that is based around recurring self-assessments and barcode-based location “key-ins” in which users scan a bar code specific to a particular location (Figure 1). Users are assigned a status of CLEAR or NOT CLEAR and are then provided personalized recommendations based on their risk and their location. A thorough detailing of the development, implementation, and utility of the app for contact tracing is shown elsewhere, but briefly: over the duration of the pilot study, 45 unique accounts were created, 227 self-assessments were performed, and users performed 1410 key-ins at 48 unique locations (out of a possible 71 locations) [29].

At the conclusion of the previously described MyCOVIDKey pilot study, we analyzed aggregate and individual app use data, and we also asked our users to provide feedback on their experience with the app. In this manuscript, we provide an analysis of these use statistics and the user feedback; we also describe the subsequent adjustments we are making to improve the app. We present this information so that public health officials who are preparing to implement digital contact tracing tools and software developers who are building them can learn from our users and their experience.

## Methods

### Institutional Review Board Approval

This study was reviewed and approved by the Vanderbilt University Institutional Review Board (#200976; June 1, 2020).

### Pilot Study Design

The MyCOVIDKey pilot study ran from June 17 to July 29, 2020, and was centered around a series of interconnected science and engineering buildings on Vanderbilt University’s campus. During this phase of the COVID-19 pandemic, most work was being conducted remotely, with the exception of research that required the researcher’s physical presence on campus.

Anyone over 18 years of age with an internet-connected mobile device was eligible to participate. To recruit participants, potential users were informed of the ongoing pilot by flyers posted around the participating buildings and by two recruiting emails that were distributed through the department mailing list to faculty, staff, and students. At the end of the first week, an email was sent to department-wide email lists and the flyers were updated in Stevenson Center to announce the introduction of a weekly raffle for a US $20 Amazon gift card. A second email and an update to the posted recruiting flyers were deployed near the end of the fourth week to the same email lists, detailing an increase in the weekly raffle prize from US $20 to $45 and the addition of a second prize of a $15 gift card.

To incentivize participation, weekly raffles were performed in which the number of entries for each user correlated to that user’s app use. Briefly, users were awarded 10 points for each self-assessment performed and 1 point for each key-in; there was a cap of 30 points per week for each category, limiting users to a maximum of 60 entries into the raffle (3 self-assessments and 30 key-ins per week), and the number of points reset at the start of each week. Users could view their individual statistics and accumulation of raffle points within the web app. A modal pop-up window that displayed the user’s most frequently keyed-in locations and weekly points obtained toward the raffle could be viewed by clicking the “See Your Stats” button on the home page. Users could view box plots generated for both average daily and all-time key-ins versus average weekly and all-time scans, allowing them to compare their use to the aggregate and anonymous data of the MyCOVIDKey user base. A pop-up window was available to describe the point system toward the raffle.

### Paradata Collection and Analysis

The term *paradata* in this manuscript refers to collected data that indicate how users engaged with the app. This information includes timestamped user-performed actions and events, and it describes the process by which users interact with the MyCOVIDKey site. An in-house paradata library was built to collect data on app use. The paradata library was built using JavaScript and enabled “behind-the-scenes” data collection using AJAX, the commonly used asynchronous HTTP request library. Each time a page was loaded or a button was clicked, the following information was sent asynchronously (without blocking the user experience on the front end) to our database: timestamp, user action, user ID (if the user was authenticated with a valid username and password), the user’s current PHP session ID, the page that the action occurred on, the user’s IP address, and the user’s device and browser information. When users were authenticated, their paradata could be associated with other user feedback and actions (ie, results of screenings and key-ins). 

### Postpilot Survey

Near the conclusion of the study, participants were asked to voluntarily provide feedback on their experience with the MyCOVIDKey app. All individuals who consented and participated in the MyCOVIDKey on-campus pilot study and who provided a verified email address were invited to participate in the postpilot survey. A custom survey was hosted on Research Electronic Data Capture (REDCap), a secure research electronic database, and an individual, nonpublic link was provided by email to all registered MyCOVIDKey users at the end of the 6-week trial period [30,31]. Data entered on the survey webpage were stored directly on the REDCap server. The survey totaled 59 questions across eight sections, and users could refer to the previous pages to review or change their answers until they submitted the survey. The eight survey sections included demographics (5 questions), COVID-19 testing history (3 questions), system usability scales (10 questions per feature, 30 total questions), impressions of MyCOVIDKey (12 questions), impressions of digital contact tracing tools and features (6 questions), and open-response questions specific to MyCOVIDKey (3 questions). Demographic data included age, gender identity, race, and on-campus role (eg, student, postdoctoral researcher, faculty, or staff). Usability was measured using a System Usability Scale (SUS) [32]. The SUS consisted of 10 statements, such as “I found MyCOVIDKey unnecessarily complex,” which were then ranked using a 5-point Likert scale in which respondents were asked to what degree they agreed with the statements (strongly disagree to strongly agree). The SUS was used to assess the perceived usability of the MyCOVIDKey app as a whole and the key-in and the self-assessment features of the app independently. The SUS was scored by converting each answer to a score from 0-4, summing the total responses for each question, and then multiplying the total by 2.5. This produces a score from 0-100; 68 is considered a benchmark score for usability, and scores lower than this value are considered to indicate below-average usability. Impressions of MyCOVIDKey were also measured on a 5-point Likert scale, where participants responded to phrases such as “I found it easy to take screenings every two days” or “Using MyCOVIDKey positively impacted my feeling of safety on campus.” All phrases were positively coded to ensure consistent composite scores for all questions. To determine impressions of general contact tracing and digital contact tracing tools, a binary yes/no system was implemented to determine general user impressions regarding the importance (eg, “Do you think contact tracing is important?”), effectiveness, security, ease of use, and time and effort costs of contact tracing interventions. The final section of the survey encouraged users to fill in free-response questions relating to their personal MyCOVIDKey experiences as well as suggestions for the development team to improve usability. To encourage participation, participants who completed the survey were entered into a raffle for a US $50 Amazon gift card.

### Data Analysis

At the conclusion of the study, all paradata were exported from the MySQL database. Similarly, all survey responses were exported from REDCap. Distributions were analyzed using box plots, violin plots, and custom Likert-style plots. Linear regressions were performed to analyze user sign-up data. All analytical and quantitative statistical analysis was performed with statistical packages in Python (ie, StatsModels, NumPy, SciPy). All data visualizations were generated in Python using common numerical plotting packages (ie, matplotlib, Seaborn).

## Results

### Paradata Analysis

#### User-Aggregated Paradata

**Figure 2:**
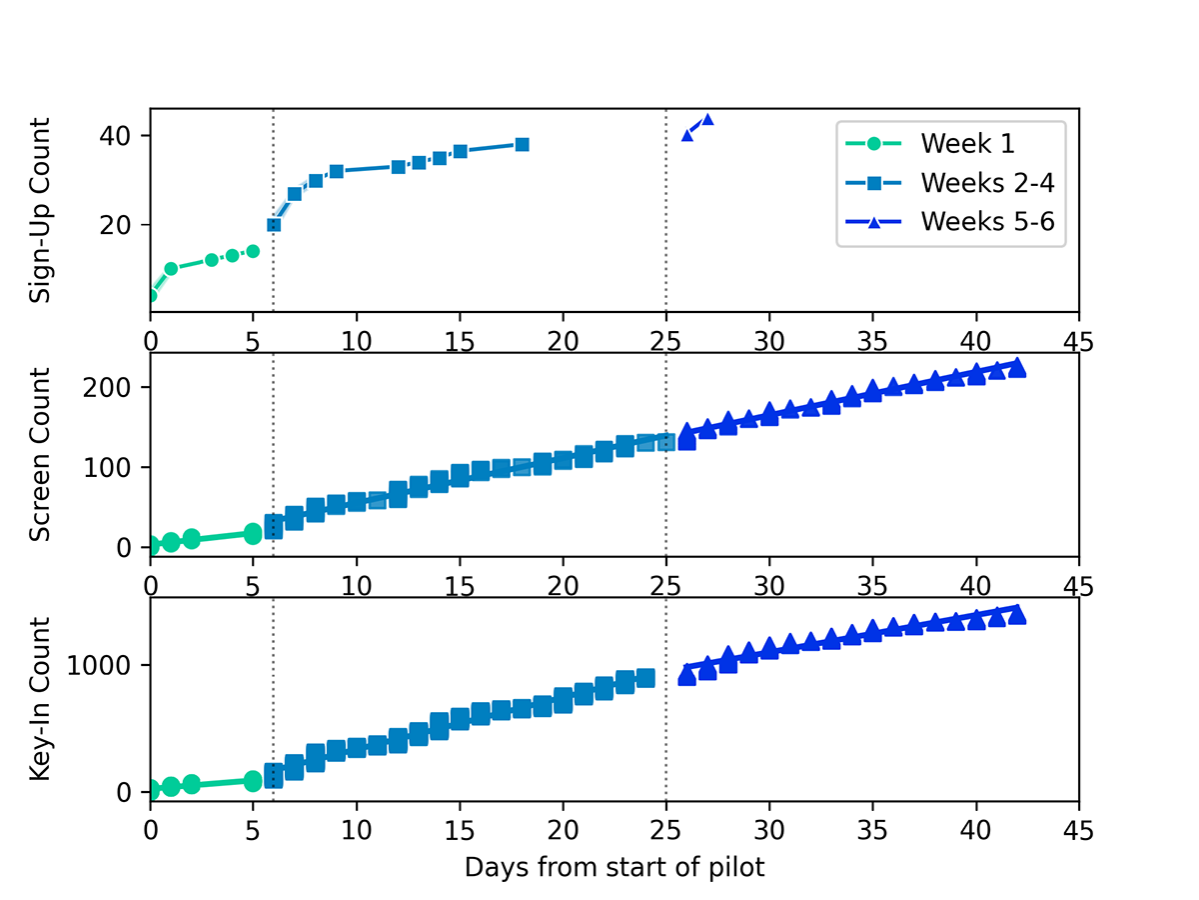
The user signups, screenings, and key-ins for the MyCOVIDKey app over three time periods during the pilot study: week 1, weeks 2-4, and weeks 5-6. The vertical dashed lines at days 6 and 25 represent days on which recruiting emails were distributed.

In the first week, organic growth of the user base quickly plateaued (Figure 2). The MyCOVIDKey user base grew organically to 14 users in the first week of use through signup flyers posted throughout the participating buildings. We observed that the first recruiting email had the most substantial impact on new user signups, while a second recruiting email was less effective (Figure 2 (top)). An additional 6 user accounts were created on the day that the first recruiting email was sent, and the user base reached 32 accounts by day 9 of the pilot and 38 users by day 18, after which it remained constant for 8 more days. The second recruiting email had a more limited effect, adding only 7 users over the course of the next week.

The self-screen and key-in counts relative to the launch date of the web app are reported in Figure 2 (center and bottom) and summarized in Table 1. In the first week, 2.85 self-assessments per day were completed across all users. This number increased to 5.58 screenings completed per day after the first promotional email and remained approximately constant (5.39 per day) after the second promotional email.

Key-ins saw a similar uptick in use after the first promotional email, increasing from 13.1 key-ins per day to 40.8 key-ins per day. After the second email, the number of key-ins per day decreased to a rate of 29.2 per day.

**Table 1:** Linear regression parameters for screenings and key-ins over each of the three study periods.

Slope and study week		Screens per day	*r*^2^ (SE)
**Screening slope**			
	1	2.85	0.944 (0.228)
	2-4	5.58	0.989 (0.078)
	5-6	5.39	0.991 (0.077)
**Key-in slope**			
	1	13.1	0.929 (0.531)
	2-4	40.8	0.993 (0.170)
	5-6	29.2	0.970 (0.325)

#### Paradata to Determine Incentive-Driven App Use

**Figure 3:**
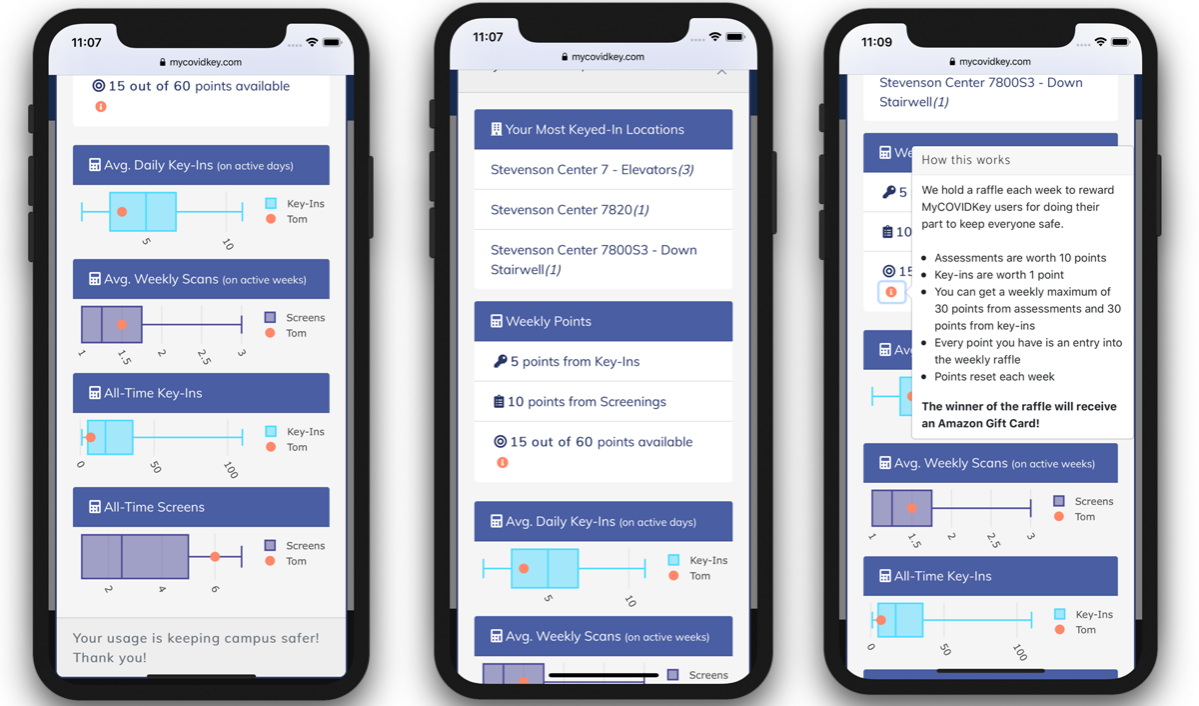
Screenshots of the user statistics modal in MyCOVIDKey. Users were presented with statistics comparing their app use to that of the rest of the userbase along with their progress toward the maximum number of raffle points allowed each week.

We evaluated the user-generated paradata to see if there were any users that would be considered “high-score seekers”—users who are primarily interested in reaching the maximum number of points (Figure 3). Specifically, we compared the number of scans and key-ins for each user with the number of times that user clicked on the “See Your Stats” modal button to view their number of entries in the weekly raffle. The paradata showed that the number of views of the statistics modal for each user generally correlated with increased use of the other app features (key-ins, Multimedia Appendix 1 top; self-assessments, Multimedia Appendix 1 bottom). This trend was also generally seen when comparing the number of logins for each user (color of markers). There are two obvious outliers on different ends of the analysis: (1) a user who viewed their statistics substantially more than twice the number of times of the next highest user (ie, used the screening feature more often than average) but who did not key in frequently; and (2) a user who had nearly double the number of key-ins as the next highest user, performed relatively few self-assessments, and viewed their stats only a few times.

### Event Statistics

Throughout the 6-week pilot study of MyCOVIDKey, paradata were collected and analyzed to better understand the use and usability of the contact tracing platform. From the paradata, 45 users logged a total of 1270 unique sessions and used 114 distinct browser/mobile device combinations. The time required to create a MyCOVIDKey account was measured from the first presentation of the account creation page to the time that the completed user registration was recorded in our database. The entire account creation process took users an average of 2.30 minutes (SD 2.07) to complete. Once users created an account, they were asked to complete an initial self-assessment. Each of the self-assessments had an expiration lifetime of 48 hours, at which point the user was required to complete another assessment prior to accessing other features in the app. On average, the time to complete a screening was 18.22 seconds (SD 20.04) (Multimedia Appendix 2, top), with an average time of 3.83 days (SD 4.23) between screenings (Multimedia Appendix 2, center). This was expected, as each screening remained valid for 48 hours. Measuring from the time that a user launched the modal to scan a barcode to the time that the pop-up window was closed, key-in events had a mean duration of 75.30 seconds (SD 97.89) (Multimedia Appendix 2, bottom). Removing any instances in which the modal was presented and the user did not scan a barcode at a location, these key-in events included an average of 3.17 key-ins (SD 4.59) per time that the modal was launched. This indicates that most users scanned barcodes at multiple locations within the same session. These disparities were then compared for each individual user, and the results showed that individual users mostly mirrored the aggregate distributions (Multimedia Appendix 3).

**Figure 4:**
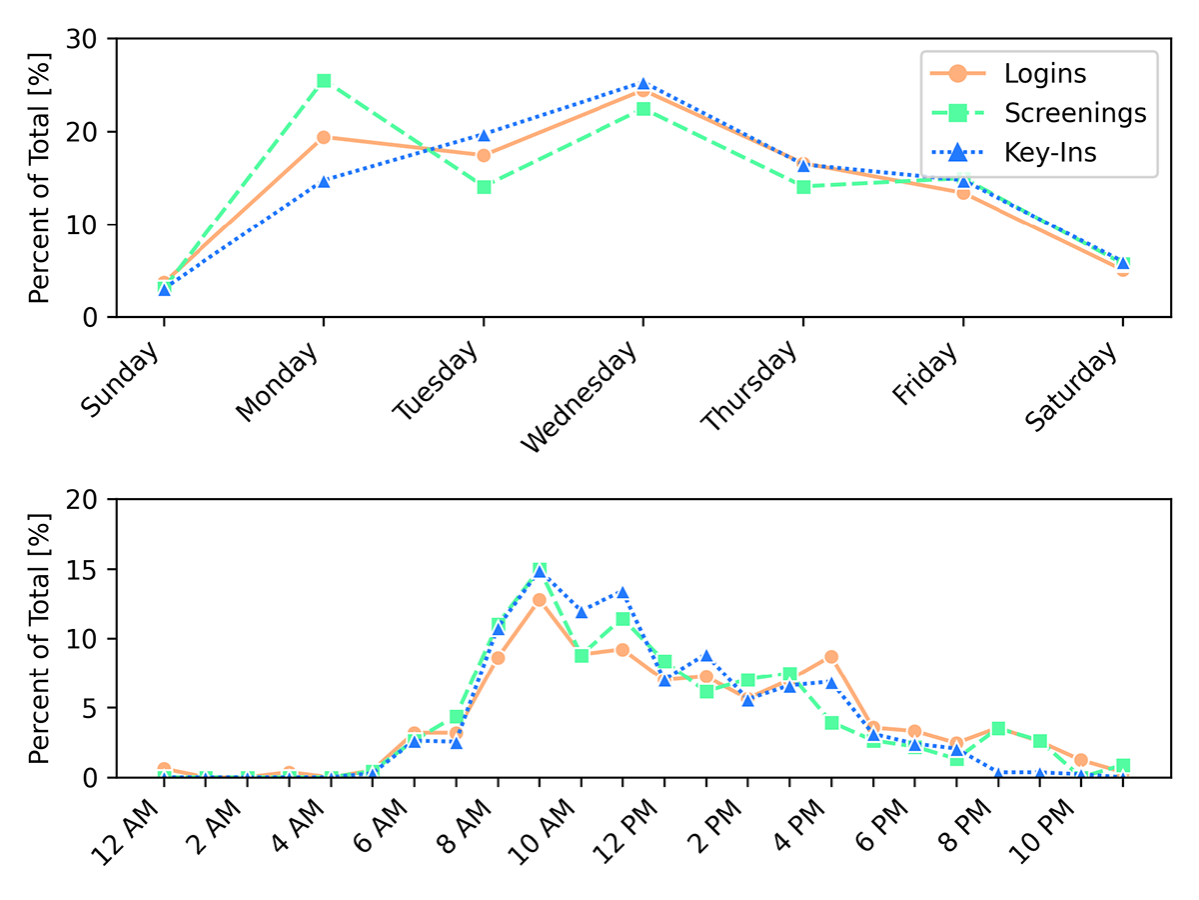
Use of the MyCOVIDKey app by (top) time of day and (bottom) day of week. Time of day is user local time (CST). Total values for reference: 814 logins, 227 screenings, and 1410 key-ins.

During the study, the Stevenson Center Complex operated on a limited access basis; graduate students and faculty maintained staggered schedules, which varied from hourly shifts to alternating days for each research group. Thus, several people were entering and exiting the building at various points throughout the day. Every login, key-in, and screening was grouped by the day of the week and time of day that each was performed, and the distribution of percentages is shown in Figure 4. Both logins and key-ins followed a similar trend in which they each exhibited an increase until a midweek peak. Notably, activity was minimal during the weekend, as expected. The highest percentage of screenings (58/227, 25.6%), on the other hand, was performed on Monday. Once a user finishes a screening, the CLEAR or NOT CLEAR status remains for a period of 48 hours. Therefore, if a screening was completed on Monday, the user would not have to take another until Wednesday, resulting in a decrease of screenings on Tuesdays and another increase two days later on Wednesday.

Similarly, each login, key-in, and screening was sorted by the time of day at which it was accessed (Figure 4). After peaking at 9 AM, logins remained relatively consistent between 10 AM and 4 PM, followed by a sharp decrease through the late afternoon and evening. Consistent logins were expected, as the server automatically logged users out for security reasons after 20 minutes of inactivity. After that, a new login was required to key in at a new location or complete a screening. More than half of all logins (61.5%), key-ins (72.4%), and screenings (68.28%) occurred before 1 PM, and most of them occurred from 9 AM to 10 AM (logins, screenings, and key-ins: 12.78%, 14.98%, and 14.89%, respectively). 

#### User Demographics and COVID-19 Testing

Of the 45 MyCOVIDKey users during the pilot period, 26 (58%) completed the postpilot survey; 4 users (9%) started the survey but did not complete it. Our survey respondents were primarily White (24/30, 80%), female (20/30, 67%), and aged 20-30 years (22/30, 73%) (Table 2). Three quarters of our users (23/30, 77%) identified themselves as graduate students. The high proportion of graduate students enrolled during the pilot period was expected, as Vanderbilt University’s reopening policies emphasized remote work, which was more readily achievable for faculty and administrative staff than for graduate students involved in laboratory research.

**Table 2:** Survey respondents’ demographic characteristics and campus roles (N=45).

Characteristic		Value, n (%)
**Survey status**		
	Sent	45 (100)
	Complete	26 (58)
	Incomplete	4 (9)
	Not started	15 (33)
**Gender**		
	Female	20 (67)
	Male	10 (33)
**Race**		
	White	24 (80)
	Asian	2 (7)
	Hispanic, Latino, or of Spanish Origin and^a^ White	2 (7)
	Black or African American	1 (3)
	Asian and^a^ White	1 (3)
**Age (years)**		
	20-30	22 (73)
	31-40	6 (20)
	41-50	1 (3)
	>60	1 (3)
**Campus role**		
	Graduate student	23 (77)
	Faculty member	6 (20)
	Postdoctoral researcher	1 (3)

^a^Users selected multiple checkboxes.

Although the self-assessment feature provided users with a symptom selection that could indicate potential infection with SARS-CoV-2, users were not asked to provide any information about their experiences or results from COVID-19 diagnostic testing within the MyCOVIDKey app. In the postpilot survey, we asked users if they had received diagnostic testing, and if so, to provide the results of this diagnostic testing (Table 3). More than one-third (11/30, 37%) of our users were tested for SARS-CoV-2 throughout the pilot, with 30% (9/30) of the users being tested only one time and 7% (2/30) of the users being tested twice. One user (1/30, 3%) indicated that they had tested positive for SARS-CoV-2 during the pilot period.

**Table 3:** Survey responses related to COVID-19 testing during the pilot period (n=30).

Variable			Value, n (%)
Not tested			19 (63)
Tested			11 (37)
**Number of tests**			
	1	9 (30)	
	2	2 (7)	
**Test result**			
	Positive	1 (3)	
	Negative	10 (33)	

### Postpilot Survey

#### System Usability Scores

**Figure 5:**
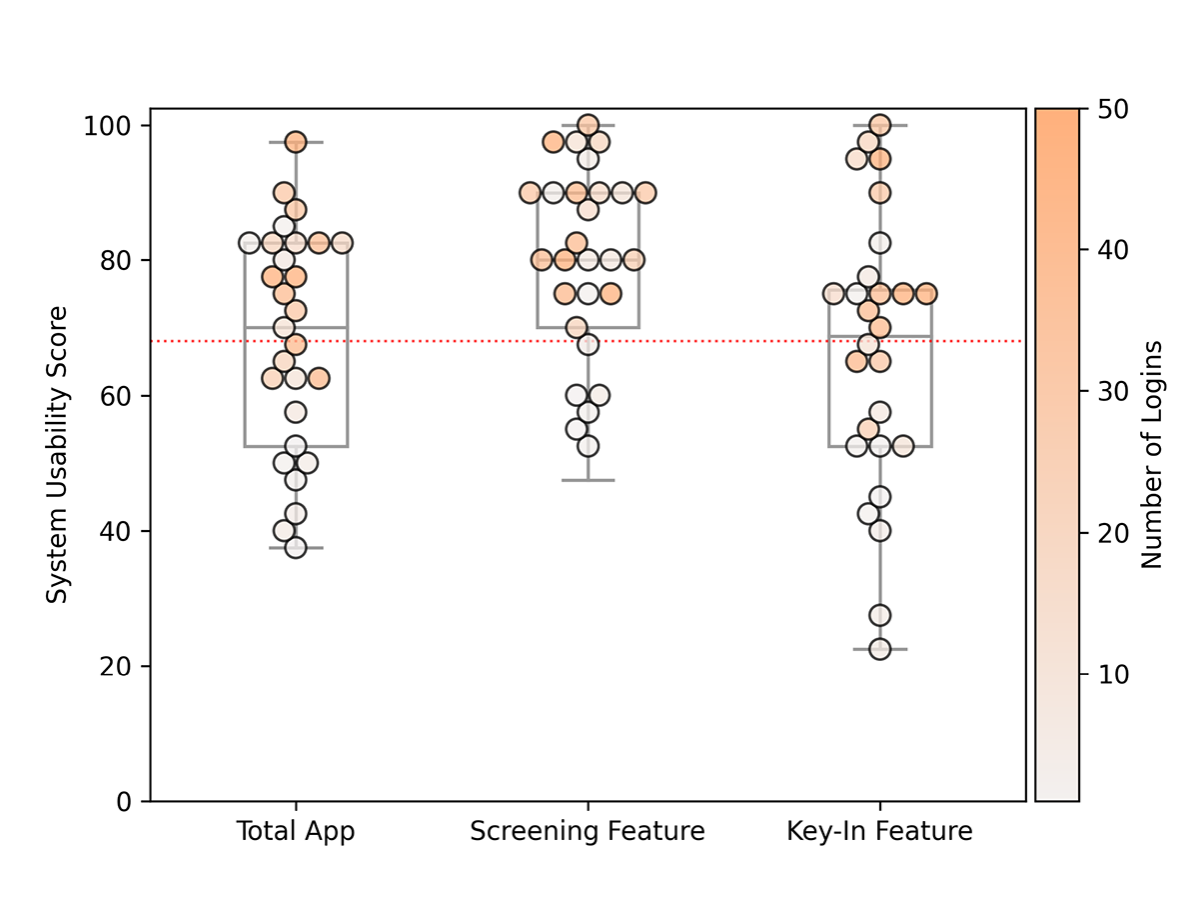
System Usability Scale scores for the total MyCOVIDKey app, the screening feature, and the key-in feature. The threshold for acceptable usability of 68 is represented with a dashed horizontal line. The markers represent the scores provided by individual users, and the intensity of the color correlates to the number of logins for that particular user. The maximum color intensity indicates users with more than 50 logins.

Users were also asked to provide their impressions on the MyCOVIDKey app as a whole as well as the self-assessment and key-in features individually (Figure 5). Using an SUS score of 68 as the threshold of acceptable usability (red dotted line in Figure 5) [32], the app as a whole can be considered to have adequate usability (SUS 70). The screening feature easily passed this metric, with a median score of 80.0 and a bottom quartile score of 70.0. The key-in feature appeared to be more polarizing in its usability; the range of its SUS scores was much larger, with a minimum and maximum score of 22.5 and 100, respectively. Although it still surpassed the threshold for usability, the key-in feature had the lowest top quartile score (75.6), median score (68.75), bottom quartile score (52.5), and minimum score. Each individual user’s SUS score was compared to that user’s number of logins (Multimedia Appendix 4, top), self-assessments (Multimedia Appendix 4, center), and key-ins (Multimedia Appendix 4, bottom). For the app as a whole and for the individual features (self-assessments and key-ins), there were positive correlations between more frequent use and higher SUS scores.

#### User Preferences for Contact Tracing Apps

**Figure 6:**
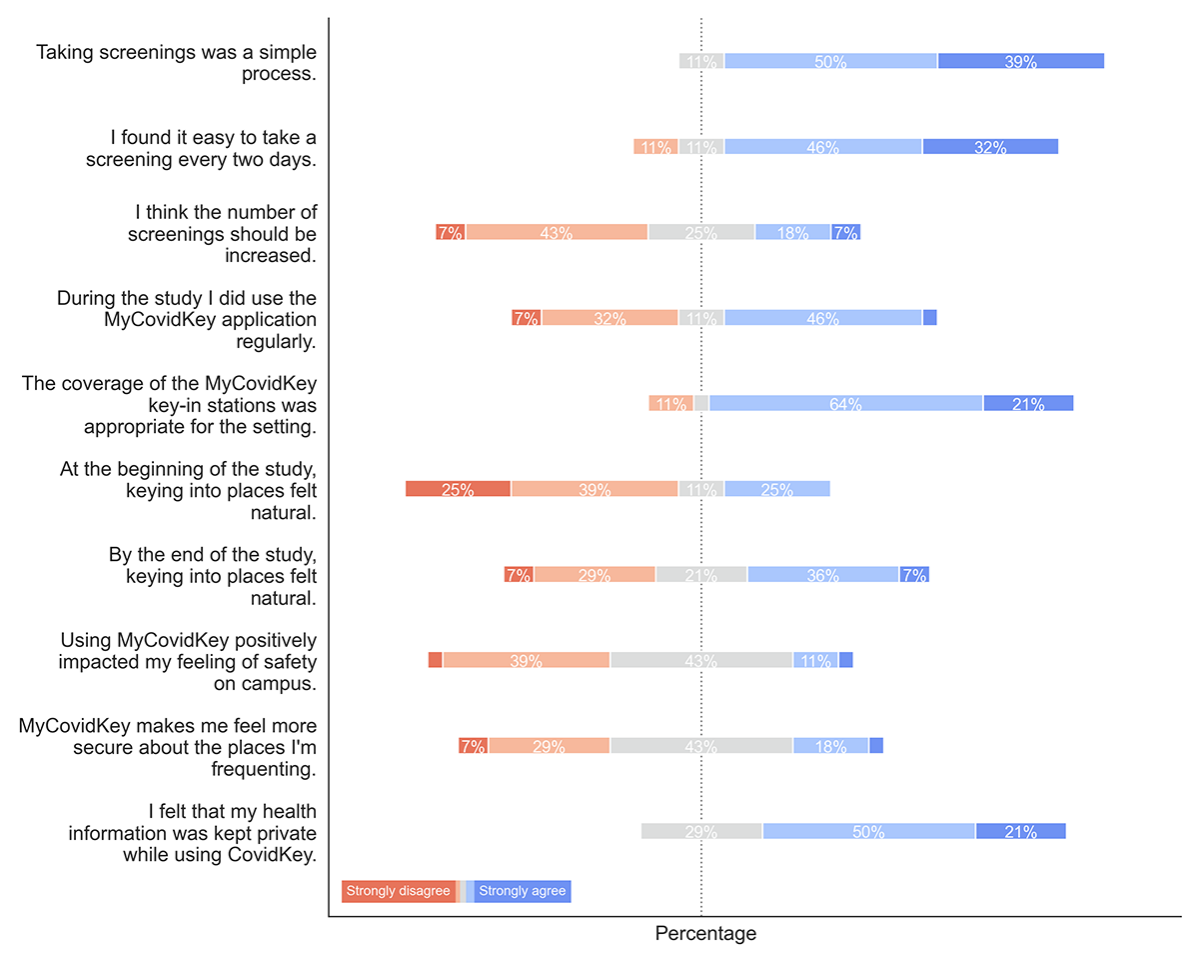
The distributions of responses to specific questions about MyCOVIDKey (n=28).

In addition to the SUS-related questions, this study focused on understanding user perceptions (Figure 6) of MyCOVIDKey using a series of questions on the Likert scale (strongly disagree: 1, to strongly agree: 5). Users strongly agreed with the statements that taking screenings was simple and easy to do every two days; however, a majority of users disagreed that the number of screenings should be increased. In general, users thought that the coverage of MyCOVIDKey key-in stations around the buildings used for the pilot study was appropriate. There was a positive shift in user perception of keying in over the course of the pilot, with 43% (12/28) of users agreeing or strongly agreeing that keying in felt natural by the end of the study compared to 25% (7/28) at the beginning. A large portion of users (12/28, 43%), were mostly ambivalent on whether MyCOVIDKey made them feel safer around campus or at locations that they were visiting, with slightly more respondents disagreeing with those statements than agreeing. Over 70% of users (20/28, 71%) agreed or strongly agreed that they felt their health information was kept private while they used MyCOVIDKey.

When these distributions were separated into groups based on the SUS score that the user provided (Multimedia Appendix 5) and the number of times the user logged in (Multimedia Appendix 6), the distributions across the Likert scale were mostly similar. Users who used the app less frequently mostly self-identified as less frequent users (Multimedia Appendix 6), and this distribution was also skewed toward less positive ratings of the app (Multimedia Appendix 5). There are several other notable differences; users who logged into the app more frequently and gave it a higher SUS score had a more skewed distribution than those who logged in less frequently and gave lower SUS scores. For instance, more users who gave SUS scores below the threshold of usability disagreed or strongly disagreed with the statements that taking screenings was a simple process, it was easy to take a screening every two days, the coverage of MyCOVIDKey key-in stations was appropriate, and that it felt natural keying in to places at the end of the study. This analysis held true when the users were separated as logging in more or less than the median number of logins. Users who rated the app higher and users who logged in more frequently had more positive impressions of safety.

**Figure 7:**
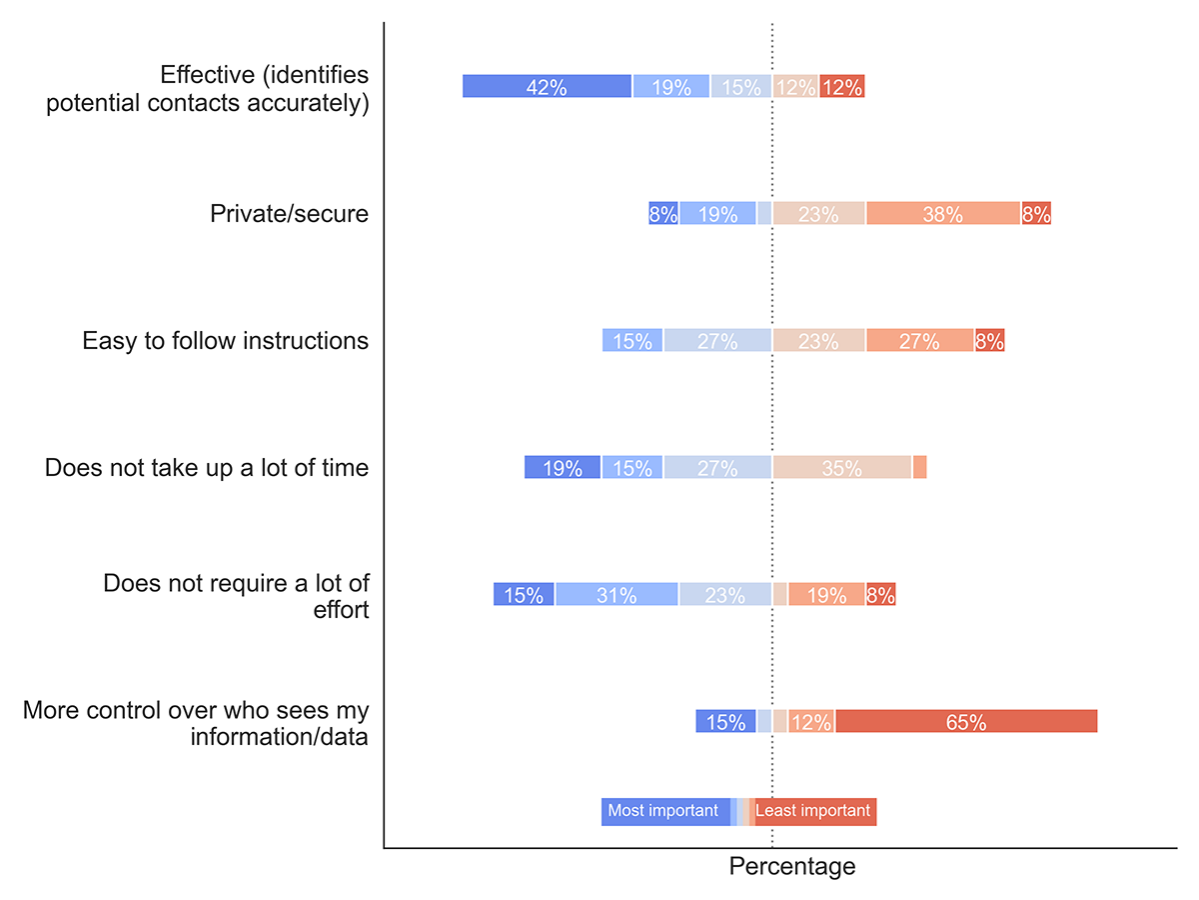
A diagram depicting how MyCOVIDKey users ranked the importance of the features of the app. The questions asked the users (n=26) to identify the most important (blue) and least important (red) features of the app.

Although users were not explicitly asked to compare MyCOVIDKey to other contact tracing approaches, we recognize that contact tracing is a new phenomenon to the general public; thus, we sought to understand which features users prioritized (Figure 7). The study population strongly indicated that contact tracing effectiveness (ie, the platform accurately identifies potential contacts) was the most important value proposition, as 42% of users (11/26) ranked it as the most important trait of a contact tracing tool. Users next valued minimization of effort and time, where 46% (12/26) and 34% (9/26) of users ranked these traits as either the most or second most important, respectively. Conversely, the MyCOVIDKey users appeared to deprioritize privacy, given the trade-off between the other characteristics. Surprisingly, 65% (17/26) of the survey respondents indicated that having more control over who sees their information or data was the least important feature.

User preferences were largely the same across users who gave MyCOVIDKey a lower SUS score (Multimedia Appendix 7) and across infrequent users (Multimedia Appendix 8). Some interesting deviations from this pattern are that more users who rated the app below the usability threshold ranked privacy as less important and ranked minimal effort as their most important preference. In contrast, users who logged into MyCOVIDKey more frequently more often ranked minimizing effort as their lowest priority.

### Direct User Response to MyCOVIDKey

Although the majority of the poststudy survey questions enabled users to select from a predefined set of answers, users also provided open-ended responses regarding their opinions on the best and worst parts of MyCOVIDKey and on how the app could be improved. The open-ended responses are aggregated in Table 4.

**Table 4:** Aggregated user responses to open-ended questions.

Category and response			Value, n (%)
**Best part of MyCOVIDKey**			
	Total responses	18 (100)	
	Useful tool, good for contact tracing	6 (33)	
	Accessible/good locations for barcodes	3 (17)	
	Easy to use/simple	3 (17)	
	Tracks statistics/game-like	2 (11)	
	Minimal time required	2 (11)	
	Scanning worked well	2 (11)	
	Self-assessments	2 (11)	
	Gift card incentives	1 (6)	
**Worst part of MyCOVIDKey**			
	Total responses	19 (100)	
	Web browser	10 (53)	
	Key-in did not always work as expected	6 (32)	
	Difficult to use when carrying things	3 (16)	
	Automatic logout	3 (16)	
	Effort required	2 (11)	
	Unsure who sees information	1 (5)	
	Frequent self-assessments	1 (5)	
	Number of steps to get to key-in window	1 (5)	
	Unclear instructions	1 (5)	
	Unsure if others are using frequently/effectively?	1 (5)	
	Expectation of receiving notification if someone tested positive for SARS-CoV-2	1 (5)	
**Ways to improve MyCOVIDKey**			
	Total responses	20 (100)	
	Make it a native app	12 (60)	
	Open directly to scanner, faster scanning	4 (20)	
	Integration with other location services, self-report locations visited	4 (20)	
	Offline mode	2 (10)	
	More key-in locations	2 (10)	
	More transparency on how information is collected and shared	1 (5)	
	Clearer instructions for use	1 (5)	
	More use statistics	1 (5)	
	Option to self-report positive test result	1 (5)	

In the “best part of MyCOVIDKey” open-ended responses, users indicated that they believed the tool had purpose and was a suitable option for contact tracing. Other responses noted that the platform was simple, was accessible at expected locations, worked well, and had recurring self-assessments. There were also two mentions of statistics/game-like mentality, and one mention of the gift card incentives. From the “worst part of MyCOVIDKey” and the “improve MyCOVIDKey” responses, there is a clear directive to build the platform as a native app (10 of 19 responses identified the web browser in the “worst” section, and 12 of 20 responses in the “improve” section asked for it specifically to be made into an app). This appears to be an umbrella response for many users, as users also noted room for improvement with regard to the automatic logout feature, an open direct to scanner feature, offline mode, and integration with other location services. A few users indicated that it was difficult to use MyCOVIDKey when carrying things, which is a now-obvious problem for chemistry graduate students working on experiments in multiple laboratories. These users, and others, noted that the ability to integrate with other location services or to self-report locations they had visited would be a helpful remedy. Although 2 of 18 users shared that scanning worked well, some users (6/19) did have unexpected issues with the key-in feature.

## Discussion

Contact tracing is poised to play a large role in the strategic preparedness and response plan during the remainder of the COVID-19 pandemic in the United States. With evidence of broad asymptomatic transmission, identifying individuals who may have been exposed and providing them with proper testing and isolation is an essential means to slow the spread of disease. As case numbers and deaths continue to increase rapidly, resource-intensive manual contact tracing can be augmented with digital tools that can efficiently identify those at risk of exposure. We developed MyCOVIDKey to provide an alternative to other digital contact tracing solutions that use constant GPS and Bluetooth monitoring and pose potential privacy concerns. In this work, we focused on analysis of user-generated paradata and a postpilot survey to understand user impressions and develop a roadmap for improvements.

### Usability of MyCOVIDKey

SUS scores were used to evaluate the platform as a whole as well as its two main features. An SUS presents a simple and objective tool to evaluate basic usability and identify areas for improvement. The median SUS score for the MyCOVIDKey app overall was above the threshold for acceptable usability, albeit close to it. Although the key-in feature was above this threshold, it was closer to the cutoff, and the survey respondents expressed more grossly divergent opinions about this feature. In contrast, the recurring self-assessments scored well, with more than 75% of the feature-specific SUS scores deeming its usability to be acceptable. In general, users who more frequently participated in the study through app use reported higher SUS scores on average for each feature of MyCOVIDKey.

In addition to the SUS questions, specific sections of the survey were focused on understanding user impressions of the two main features of the app. Survey respondents indicated that the self-assessments were easy to use, simple, and noninvasive. This was reinforced by the paradata, which showed that the majority of assessments could be completed in less than 1 minute. The responses to the survey showed that users had a more polarized opinion of the key-in feature, which also aligns well with the SUS scores and paradata.

User-generated paradata represent an important tool to understand individual and aggregate behavior within an app. Although paradata are commonly tracked and analyzed in consumer apps, their use has received considerably less attention in health care–related apps. Despite survey respondents’ criticisms of the ease of use of the key-in feature of MyCOVIDKey, we note that it was frequently used, and the paradata indicate that users were able to perform this task relatively quickly. However, the negative survey responses were strong, and the paradata may reflect the fact that a core group of users dominated the use of the app and perhaps were less critical of the feature.

### User Priorities

From our survey responses, several clear user priorities were identified. Our users indicated that their three most important characteristics of a digital contact tracing tool were (1) effectiveness at accurately tracing contacts, (2) not requiring much time, and (3) not requiring much effort. When given the opportunity to rank their preferences, users ranked control over who sees information and security as the two least important characteristics of a digital contact tracing tool. This result was surprising, considering the discourse surrounding mobile contact tracing apps; however, it was also rarely mentioned in the open response section of the survey. Additionally, this result contrasts with those in previously published studies describing attitudes toward contact tracing digital apps [26,28]. Our users, regardless of how they scored the usability of the app or how often they logged in, indicated that they felt their information was securely maintained in MyCOVIDKey. In that sense, these responses may reflect users’ opinions specifically on MyCOVIDKey in the context of the pilot study, particularly one that took place at a research institution and primarily enrolled graduate students and faculty, and may not be representative of the broader population. Furthermore, as the Department of Chemistry is a relatively small, self-contained environment, participants may have felt more comfortable regarding privacy concerns knowing that the study was occurring within their community. Indeed, concerns over privacy have been linked to larger, for-profit corporations and technology companies, and data security is typically a larger consumer concern in the event of any data breach.

### Incentivized Participation in Digital Contact Tracing

It is generally understood that digital contact tracing platforms must reach a critical user volume to be effective. Employers or educational institutions can require that their employees or students use these platforms as a condition of their employment or access to facilities; however, this requirement may be met with resentment and have a negative impact on user perception and cooperation with contact tracing teams. Regardless, we were unable to require user participation in our pilot study. In place of a mandate, after a week of moderate enrollment, we deployed a weekly raffle to encourage uptake and continued use of MyCOVIDKey. The number of entries for any given user was based upon the number of key-ins and self-assessments that the user performed that week, with a cap on each to minimize the effect of high-score seekers. Flyers advertising the pilot study were modified to announce the raffle, and a recruiting email was sent out to departmental email lists to promote the study and the raffle. In the three days after this change, the number of accounts created more than doubled, and we saw at least 2-fold increases in the rates of key-ins and self-assessments. This approximately proportional increase suggests that the increases in use rates was tied to the increase in users and was not simply due to increased use by previous users due to the raffle. Because the raffle was announced at the same time as the first marketing email, we cannot decouple the effect of one from that of the other.

After 2 weeks, the raffle prize was increased, and we announced a runner-up raffle prize to (1) avoid a drop-off in use after the July 4 holiday and (2) attempt to further increase our user enrollment. The number of new user accounts created after this change was minimal, and there was a surprising decrease in use rates (both key-ins and self-assessments per day). There are several possible explanations for this, including user signup saturation, individual work schedules, and pandemic fatigue. Regardless of the reasoning, this result does suggest that the initial incentive of the raffle was sufficient. Although 2 users noted the presence of an incentive or the gamification of the scoring system as features they enjoyed about MyCOVIDKey, the analysis of the paradata did not suggest that this was the only motivational factor in their use of the app.

It is clear that the first recruiting email, with its incentive for use, had a positive impact on user signups. The sharp increases in the screening rate and key-in rate were mostly a result of the influx of users, after accounting for the number of users active during each period of the study. The second recruiting email, even with increased incentives, resulted in modest new account signups and decreased use rates for self-assessments and key-ins.

With the number of potential users (those who were working in our study buildings throughout the pilot period) remaining constant, the lower number of signups can likely be explained by the theory of innovation diffusion. We had likely captured the early adopters and early majority, and by weeks 5-6 we were beginning to approach the late majority of users. Interestingly, the second recruiting email and the more valuable raffle prize coincided with a slight decrease in screenings per day and a more noticeable drop in key-in rate.

### Strengths and Limitations of This Study

This study is one of the first rigorous pilot evaluations performed on a digital contact tracing app. It is one of the first formal studies to investigate the usability of a COVID-19 contact tracing app with the intent of making iterative improvements. The usability analysis combines both quantitative and qualitative user feedback. This study is also one of the first studies to compare user-generated paradata to user survey responses in mobile health (mHealth) apps. This valuable tool provides unique insight into the difference between perception of usability and actual use patterns.

The primary limitations of this study include a relatively modest sample size and a narrow user demographic. These weaknesses were mostly circumstantial: social distancing requirements and “safer-at-home” orders that were in place during the study limited the number of people on campus, and our prospective participant pool was limited mainly to students and faculty within Stevenson Center, our study location. This resulted in the selection of a young and technology-savvy cohort. This does limit the overall generalizability of the study, as this demographic is potentially more comfortable with technology or currently available mHealth interventions than the broader population. Another limitation of this study is that the study was stopped on July 29, and not all users used the app for 6 full weeks prior to completing the survey.

### Guidance for MyCOVIDKey and for Other Digital Contact Tracing Apps

Based on the analysis of the paradata and user feedback received from the survey, we established a set of directions for improving the MyCOVIDKey platform. The MyCOVIDKey users, while a narrow demographic, showed strong preferences for a platform that was effective at identifying potential contacts while also minimizing the effort and time required for use. It is our hope that other developers can learn from the feedback that we received.

In general, the recurring self-assessment was favorably received by our users. It received high usability scores and positive feedback in survey responses; also, the paradata indicated that the task could be accomplished quickly. The users indicated a preference for not increasing the frequency of the required self-assessments; however, this may not be a barrier based on the approval that the feature received. As such, our focus on the self-assessment will be the addition of questions related to diagnostic testing and results. The goal of the MyCOVIDKey pilot was to rapidly deploy a solution for beta testing and to identify improvements prior to an anticipated larger rollout. Because this could be accomplished without sharing personal testing results, we made the explicit decision not to ask our users for this information. However, the benefits of integrating diagnostic results, when available, are obvious. This information has the easily recognizable utility of confirming the positivity of users that indicated symptoms in the app and, also of great importance, of removing persons of interest who test negative for SARS-CoV-2 from contact tracing queues. These questions were unnecessary during the pilot evaluation but will be critical in any broader release.

As the most frequently performed user action inside the app, we will focus substantial effort on improving the key-in feature of MyCOVIDKey. Users indicated that this was something that they wanted to be able to perform faster, with some users indicating that they would like to be able to do it directly from the home screen. Additionally, some users expressed concerns over network connectivity and how that hindered their ability to use the key-in feature. All these concerns can be readily addressed by converting MyCOVIDKey from a mobile-friendly web app to a native phone app. Although mobile-friendly web apps can often blur the lines between native and web apps, it is clear that in this instance, users have preferences that can be better met by a native app. Indeed, from our survey responses, many users explicitly stated their preference for a native app instead of a browser-based platform.

### Conclusions

Digital platforms are uniquely positioned to play a large role in contact tracing efforts during the COVID-19 pandemic. In response, we have developed MyCOVIDKey, a web-based contact tracing app, and evaluated it over the course of a 6-week pilot study. In this work, we analyzed aggregate and individual use data and compared it to user feedback from a postpilot survey. We were able to obtain quantitative data to understand how and when MyCOVIDKey was used as well as how users felt about the different components of the app. Although the app and its individual features received acceptable usability scores, this work clearly shows that users prioritize contact tracing effectiveness along with minimal time and effort requirements. This feedback provides us with a clear blueprint for how to improve our app prior to an expanded rollout as well as guidelines for other digital contact tracing efforts moving forward.

## Appendix

Multimedia Appendix 1 The number of times each user launched the modal window to view their statistics compared to the number of (top) self-assessments they performed, (bottom) key-ins they performed, and (color and size) the number of times they logged in.

Multimedia Appendix 2 The probability density distribution and quartile distributions of the duration of (top) key-in events, (middle) time between screenings, and (bottom) duration of self-assessments.

Multimedia Appendix 3 The distributions of the (top) duration of scan events, (middle) duration of self-assessments, and (bottom) time between completed self-assessments for each individual user.

Multimedia Appendix 4 System Usability Scale scores for the entire app and the number of logins for each user (top). Each user’s System Usability Scale score for the self-assessment feature of the app and the number of self-assessments that they completed (middle). Each user’s System Usability Scale score for the key-in feature of the app and the number of times that the user keyed in to a location on campus (bottom).

Multimedia Appendix 5 The distribution of responses to MyCOVIDKey-specific questions, separated into responses from users who gave the overall app System Usability Scale scores above (green, above) and below (red, bottom) the usability threshold of 68. A score of 1 reflects a response of strongly disagree, and a score of 5 reflects a response of strongly agree. Markers reflect individual responses (jitter has been artificially added to enhance visualization; only discrete integer values could be selected).

Multimedia Appendix 6 The distribution of responses to MyCOVIDKey-specific questions, separated into responses from users who logged in more (green, above) and less (red, bottom) than the median number of logins. A score of 1 reflects a response of strongly disagree, and a score of 5 reflects a response of strongly agree. Markers reflect individual responses (jitter has been artificially added to enhance visualization; only discrete integer values could be selected).

Multimedia Appendix 7 The distribution of responses to user preferences in a digital contact tracing tool, separated into responses from users who gave the overall app system usability scores above (green, above) and below (red, bottom) the usability threshold of 68. A score of 1 reflects a feature that is most important to that user, and a score of 5 reflects a feature that is least important to that user. Markers represent individual responses (jitter has been artificially added to enhance visualization; only discrete integer values could be selected).

Multimedia Appendix 8 The distribution of responses to user preferences in a digital contact tracing tool, separated into responses from users who logged in more (green, above) and less (red, bottom) than the median number of logins. A score of 1 reflects a feature that is most important to that user, and a score of 5 reflects a feature that is least important to that user. Markers represent individual responses (jitter has been artificially added to enhance visualization; only discrete integer values could be selected).

## Abbreviations

mHealth: mobile health
REDCap: Research Electronic Data Capture
SUS: System Usability Scale
