# Supplementary material for: App Use and Usability of a Barcode-Based Digital Platform to Augment COVID-19 Contact Tracing: Postpilot Survey and Paradata Analysis
Source: JMIR Public Health Surveill. 2021 Mar 26;7(3):e25859. doi: 10.2196/25859 (PMC8006896; doi:10.2196/25859)
Supplement: Multimedia Appendix 2 [file publichealth_v7i3e25859_app2.pdf]

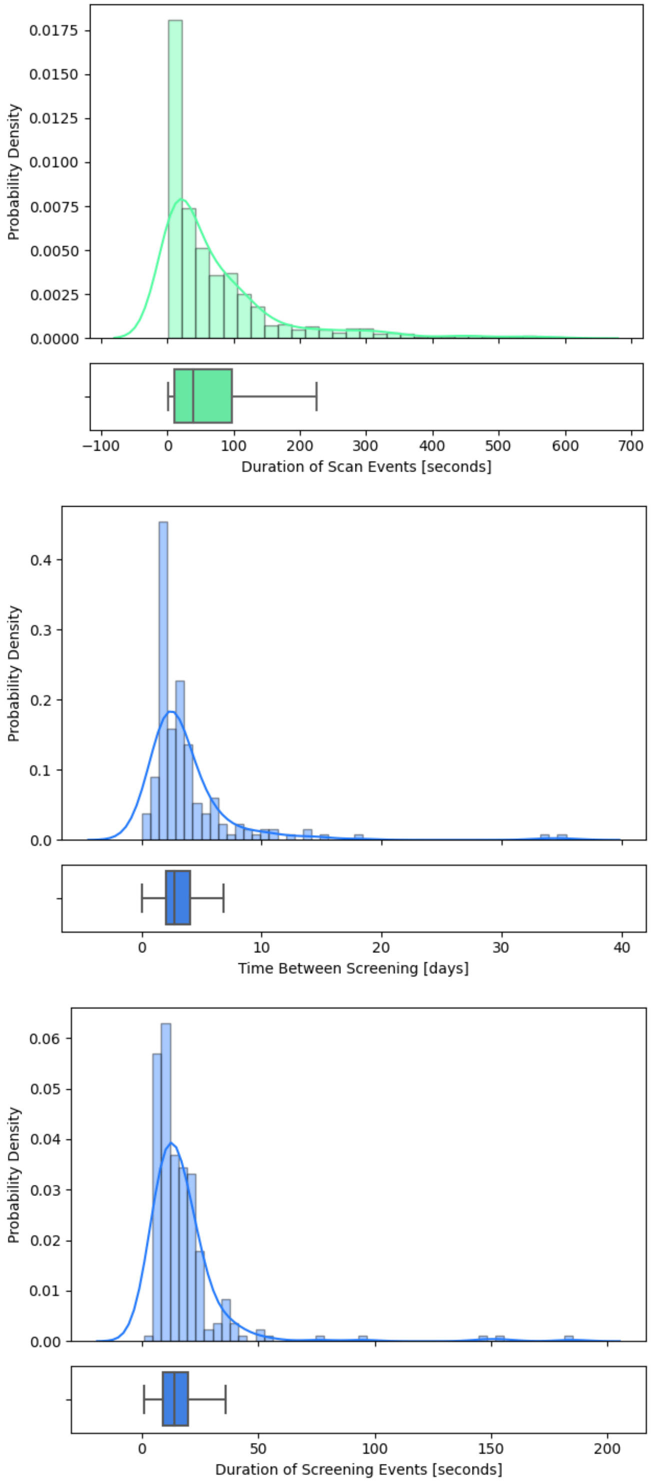


**Multimedia Appendix 2.** The probability density distribution and quartile distributions of the duration of (top) key-in events, (middle) time between screenings, and (bottom) duration of self-assessments.
