# Supplementary material for: App Use and Usability of a Barcode-Based Digital Platform to Augment COVID-19 Contact Tracing: Postpilot Survey and Paradata Analysis
Source: JMIR Public Health Surveill. 2021 Mar 26;7(3):e25859. doi: 10.2196/25859 (PMC8006896; doi:10.2196/25859)
Supplement: Multimedia Appendix 3 [file publichealth_v7i3e25859_app3.pdf]

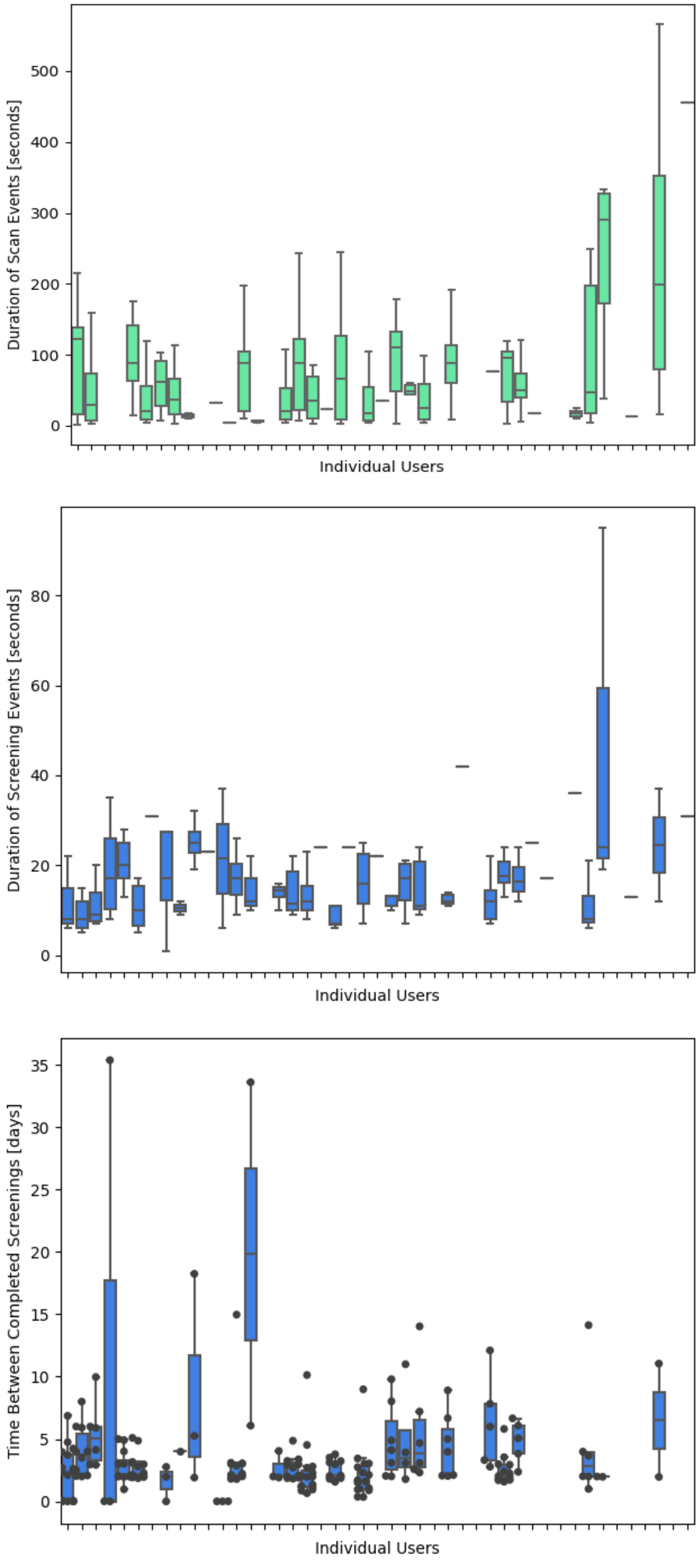

Multimedia Appendix 3. The distribution of (top) duration of scan events, (middle) duration of self-assessments, and (bottom) time between completed self-assessments for each individual user.
