# Supplementary material for: App Use and Usability of a Barcode-Based Digital Platform to Augment COVID-19 Contact Tracing: Postpilot Survey and Paradata Analysis
Source: JMIR Public Health Surveill. 2021 Mar 26;7(3):e25859. doi: 10.2196/25859 (PMC8006896; doi:10.2196/25859)
Supplement: Multimedia Appendix 4 [file publichealth_v7i3e25859_app4.pdf]

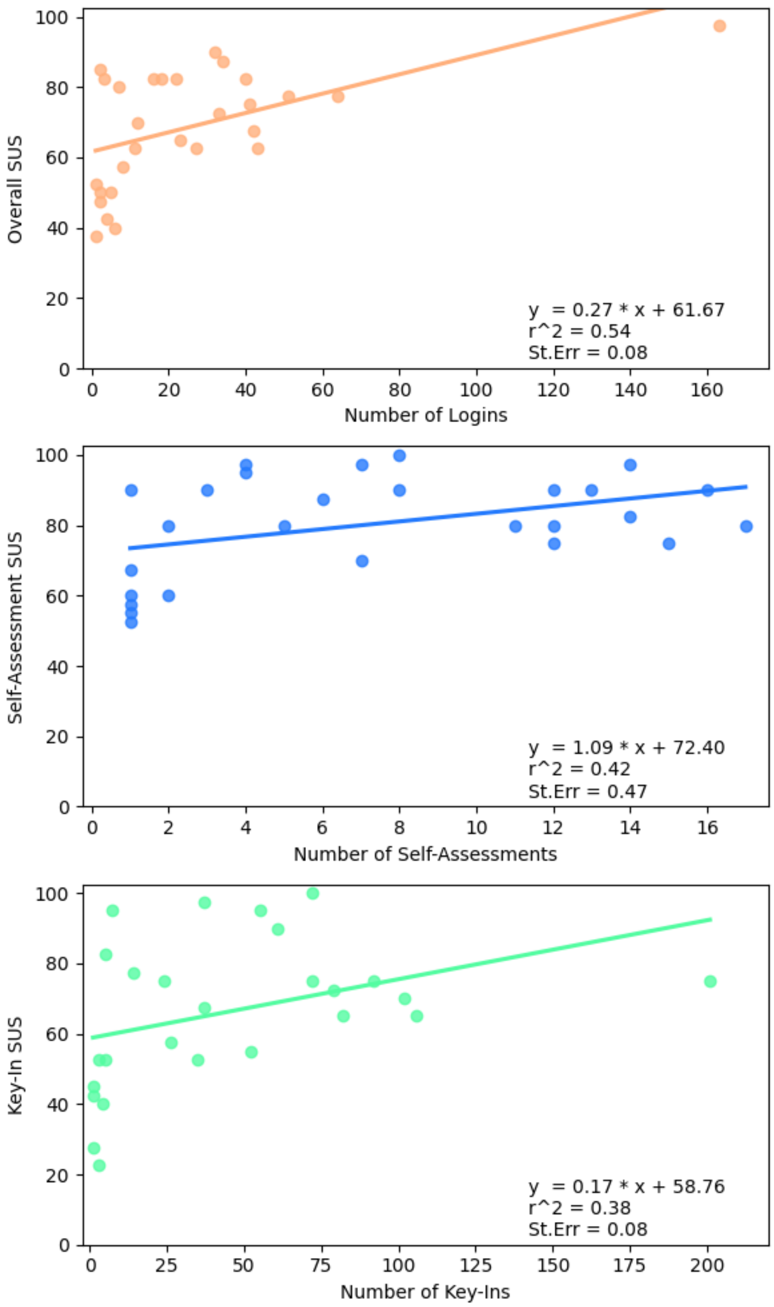

Multimedia Appendix 4. (top) Each user's SUS score for the entire app and the number of logins for that user. (middle) Each user's SUS score for the self-assessment feature of the application and the number of self-assessments that they completed. (bottom) Each user's SUS score for the key-in feature of the application and the number of times that the user keyed-in to a location on campus.
