# Supplementary material for: App Use and Usability of a Barcode-Based Digital Platform to Augment COVID-19 Contact Tracing: Postpilot Survey and Paradata Analysis
Source: JMIR Public Health Surveill. 2021 Mar 26;7(3):e25859. doi: 10.2196/25859 (PMC8006896; doi:10.2196/25859)
Supplement: Multimedia Appendix 6 [file publichealth_v7i3e25859_app6.pdf]

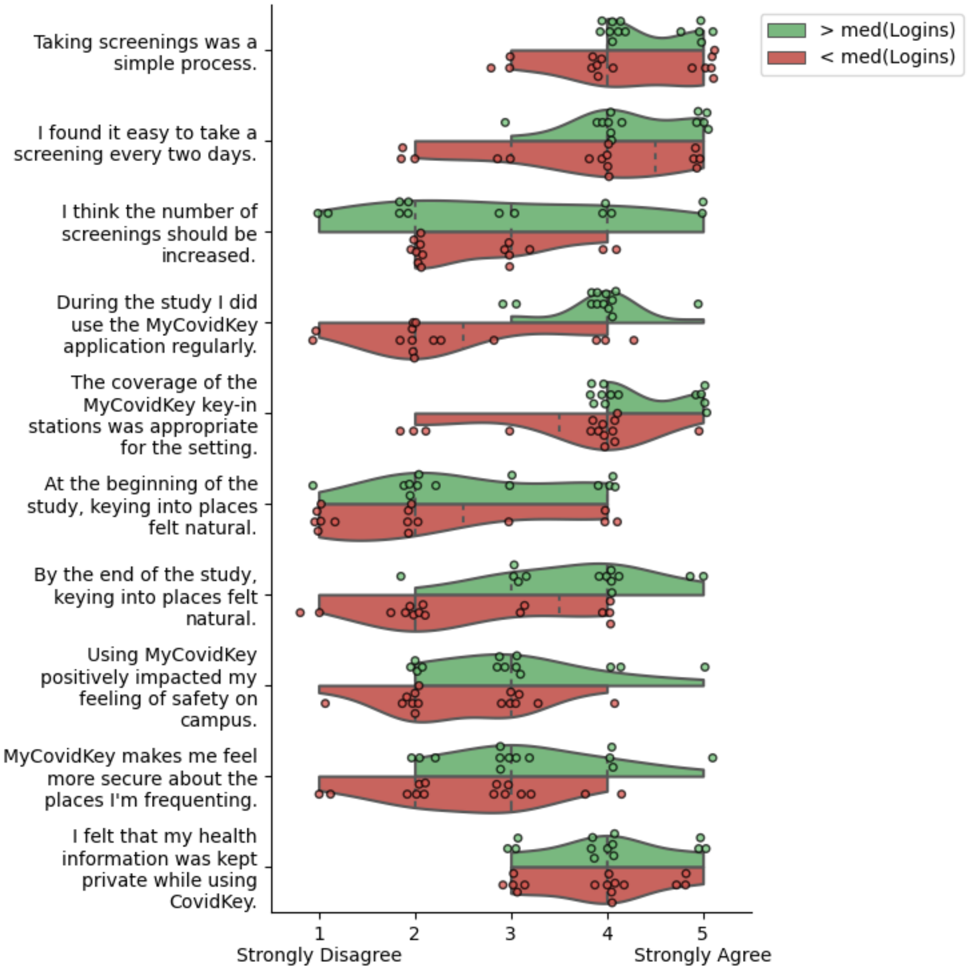

Multimedia Appendix 6. The distribution of responses to MCK specific questions, similar to Figure 7, but separated into responses from users who logged in more (green, above) and less (red, bottom) than the median number of logins. Scores of 1 reflect a response of strongly disagree, and scores of 5 reflect responses of strongly agree. Markers reflect individual responses (jitter has been artificially added to enhance visualization; only discrete integer values were able to be selected).
