# Supplementary material for: App Use and Usability of a Barcode-Based Digital Platform to Augment COVID-19 Contact Tracing: Postpilot Survey and Paradata Analysis
Source: JMIR Public Health Surveill. 2021 Mar 26;7(3):e25859. doi: 10.2196/25859 (PMC8006896; doi:10.2196/25859)
Supplement: Multimedia Appendix 7 [file publichealth_v7i3e25859_app7.pdf]

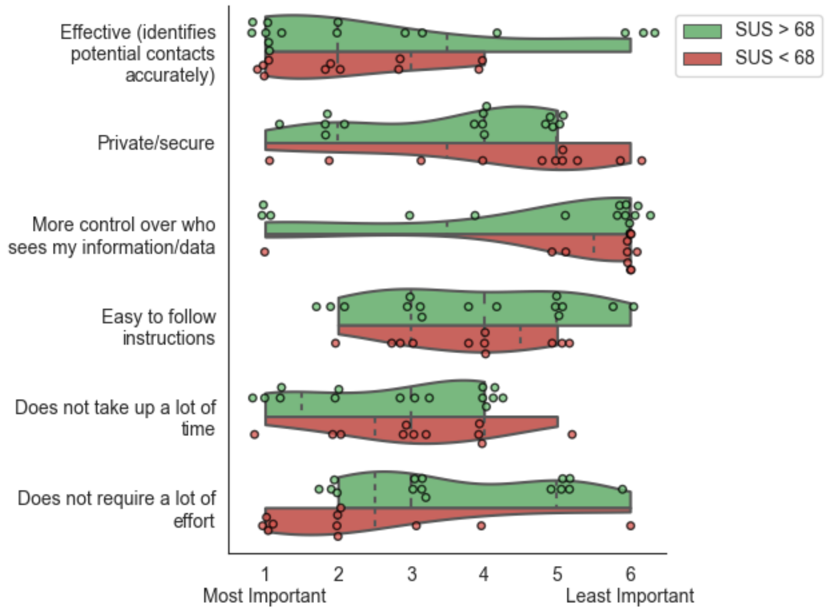

Multimedia Appendix 7. The distribution of responses to user preferences in a digital contact tracing tool, similar to Figure 8, but separated into responses from users who gave the overall application a SUS score above (green, above) and below (red, bottom) the usability threshold of 68. Scores of 1 reflect a feature most important to that user, and scores of 5 reflect a feature least important to that user. Markers represent individual responses (jitter has been artificially added to enhance visualization; only discrete integer values were able to be selected).
